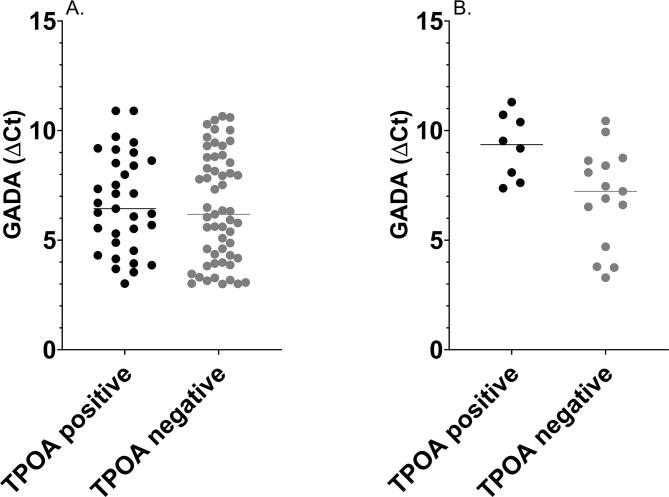# Type 1 diabetes, celiac disease, and autoimmune thyroiditis autoantibodies in population-based type 2 diabetes patients

**DOI:** 10.1016/j.jcte.2024.100367

**Published:** 2024-09-06

**Authors:** Lind Alexander, Tsai Cheng-ting, Lernmark Åke, Jendle Johan

**Affiliations:** a Department of Clinical Sciences Malmö, Lund University CRC, Skåne University Hospital, Malmö, Sweden; b Enable Biosciences Inc., South San Francisco, CA, USA; c School of Medical Sciences, Faculty of Medicine and Health, Örebro University, Sweden

**Keywords:** Type 1 diabetes, Type 2 diabetes, Celiac disease, Autoimmune thyroid disease, Autoantibodies

## Abstract

**Aims:**

The study aims were to determine autoantibodies associated with type 1 diabetes (T1D), celiac disease (CD) and autoimmune thyroid disease (AITD) in individuals living with type 2 diabetes (T2D) compared to T1D and matched controls.

**Methods:**

Individuals with T1D and T2D were randomly identified in health-care registers. Blood was collected through home-capillary sampling and autoantibodies associated with either T1D against glutamic acid decarboxylase (GADA), insulin (IAA), insulinoma antigen-2 (IA-2A), and zinc transporter 8 (ZnT8A), CD against tissue transglutaminase (tTGA) or AITD against thyroid peroxidase (TPOA) were determined in an automated, multiplex Antibody Detection by Agglutination-PCR (ADAP) assay.

**Results:**

GADA were detected in 46 % (88/191) of T1D and increased to 6.2 % (23/372) in T2D compared to 2.6 % (7/259) of controls (p = 0.0367). tTGA was low (1.1–2.6 %) and not different in between the study cohorts, nonetheless, in T1D tTGA was associated to islet autoantibodies. TPOA was more frequent in T1D, 27.1 % (53/191), compared to either T2D, 14.8 % (55/372; p = 0.0002) or controls, 14.3 % (37/259) (p = 0.0004). Overall, TPOA was more frequent in GADA positive (34.8 %; 8/23) than negative (13.5 %; 47/349; p = 0.0053) T2D individuals.

**Conclusion:**

It’s suggested that analyzing GADA and TPOA may refine the autoimmune landscape in individuals clinically classified as T2D.

## Highlights

• Autoantibodies associated with T1D, CD and AITD were determined in individuals living with T1D, T2D and matched controls.
• GADA were increased in T2D individuals, 6.2%, compared to matched controls, 2.6%.
• TPOA were increased in GADA positive, 34.8%, compared to negative, 13.5%, T2D individuals.
• Individuals classified and treated as T2D may benefit from analyzing not only GADA but also TPOA to increase the diagnostic precision.

## Introduction

Type 2 diabetes (T2D) is characterized by a low-grade inflammation that accompanies the whole trajectory of this metabolic disease, from the inception to the development of the long-term diabetes complications [1]. There is growing evidence of a list of possible “triggers” of the inflammatory response, possibly promoted by lifestyle choices, and advancing age. Individuals living with T2D show an altered number and function of their immune cells, involving both the innate and the acquired immunity [2], [3]. Autoantibodies against islet autoantigens are detected in a subpopulation of T2D patients, while some data suggests an altered function of the specific T lymphocyte populations, including T regulatory (Treg) cells [4]. Cell-mediated islet inflammation and autoreactive T cells has been associated with progressive beta cell loss in individuals with T2D to suggest a possible autoimmune stage in the disease pathogenesis [5], [6]. The presence of circulating autoantibodies in T2D was identified more than 40 years ago [7]. Islet autoantibodies in individuals with T2D is reported between 4–14 % [8] except that insulin autoantibodies (IAA) may be explained by individuals on exogenous insulin treatment [9]. The presence of primarily glutamic acid decarboxylase autoantibodies (GADA) in individuals with diabetes classified with T2D but older than 30 years of age without the need for insulin at least for 6 months characterizes latent autoimmune diabetes in adults (LADA) [10]. While autoantibodies against GAD predominate, autoantibodies to islet cytoplasm, insulinoma antigen-2 (IA-2A), and zinc transporter 8 (ZnT8A) may also be detected in these patients [11]. The aim of this study was to determine the prevalence of autoantibody biomarkers including GADA, IAA, IA-2A and ZnT8A in type 1 diabetes (T1D), tissue transglutaminase autoantibodies (tTGA) in celiac disease (CD) and thyroid peroxidase autoantibodies (TPOA) in autoimmune thyroid disease (AITD) in individuals, living in three Swedish regions, and diagnosed with T2D, and compare them to T1D individuals as well as to population-based controls.

## Materials and methods

### Subjects

The study included participants from three regions in the middle part of Sweden, i.e., Dalarna, Värmland and Örebro [12]. In 2020, these regions had a total population of 877,000 inhabitants. The respective regional health care registers were used to identify a total of 5,215 individuals living with T1D and 48,515 individuals living with T2D. A total of 180,923 population controls were identified from the Swedish tax registry. Population controls were matched to individuals with diabetes by age, gender, and postal code. A total of 3,212 individuals with T2D to be compared with 857 individuals with T1D and 1,955 matched controls were identified for study participation. The study protocol included a two-step process for participation. First, letters including study information and consents were mailed to all prospective participants. Second, when correctly signed informed consents were obtained, questionnaires and kits for home-capillary blood sampling were mailed. In total, the study included 898 participants divided in groups of 413 T2D individuals to be compared with 203 T1D individuals and 282 matched controls (Table 1).

**Table 1:** 

	Type 1 diabetes	Type 2 diabetes	Population controls	p-values
n	191	372	259	
Age, median (range)	58 (19–90)	70 (27–98)	66 (19–85)	
18–64 years, % (n)	66.5 % (127/191)	28.5 % (106/372)	44.0 % (114/259)	p^T1D_T2D^<0.0001, p^T1D_PC^<0.0001, p^T2D_PC^<0.0001
≥ 65 years, % (n)	33.5 % (64/91)	71.5 % (266/372)	56.0 % (145/259)	
Gender female, % (n)	51.3 % (98/191)	39.8 % (148/372)	48.0 % (123/259)	p^T1D_T2D^=0.0091, p^T1D_PC^=0.42, p^T2D_PC^=0.054
Body Mass Index, Median (range) (kg/m^2^)	26.1 (17.4–40.9)	28.1 (18.5–58.4)	26.0 (18.4–53.1)	
< 25.0, % (n)	41.0 % (68/166)	21.2 % (55/260)	36.8 % (78/212)	p^T1D_T2D^<0.0001, p^T1D_PC^=0.11, p^T2D_PC^<0.0001
25.0–29.9, % (n)	38.0 % (63/166)	43.9 % (114/260)	48.1 % (102/212)	
≥ 30.0, % (n)	21.1 % (35/166)	35.0 % (91/260)	15.1 % (32/212)	

Ethical approval was obtained by Swedish Ethical Review Authority (Dnr 2020-04611), informed consents were obtained from all study participants.

### Antibodies

Plasma samples were analyzed using Hamilton Microlab ADAP STAR (Hamilton, Bonaduz, Switzerland) hands-free automated liquid-handling platform. An 8-multiplex assay was developed by Enable Biosciences Inc. (South San Francisco, CA) and all reagents were obtained from the company to allow the determination of T1D (GADA, IAA, IA-2A, ZnT8A), CD (tTGA) and AITD (TPOA) autoantibodies. The Antibody Detection by Agglutination-PCR (ADAP) methodology is established on the principle of antibody agglutination to DNA-barcoded proteins, synthesis of protein-DNA conjugates was previously described [12], [13], [14], [15], [16], [17]. A previous validation study found the ADAP assay to be superior to standardized radiobinding assays for IAA and tTGA, and with a comparable performance for GADA [13]. The ADAP assay demonstrated top-tier results in the 2023 Islet Autoantibody Standardization Program (IASP) workshop for GADA (90 % sensitivity at 98.9 % specificity), IA-2A (74.0 % sensitivity at 98.9 % specificity), IAA (42.0 % sensitivity at 100.0 % specificity), and ZnT8A (70.0 % sensitivity at 100.0 % specificity).

### Statistical analyses

Frequencies of autoantibody positivity were compared between the study groups using Chi-square test and Fisher's exact test. Mann–Whitney U-tests were applied to compare continuous variables, age and body mass index. A two-sided p-value below α 0.05 was considered statistically significant. Calculations were computed using GraphPad Prism 9.00.

## Results

### T1D related autoantibodies

**Fig. 1:**
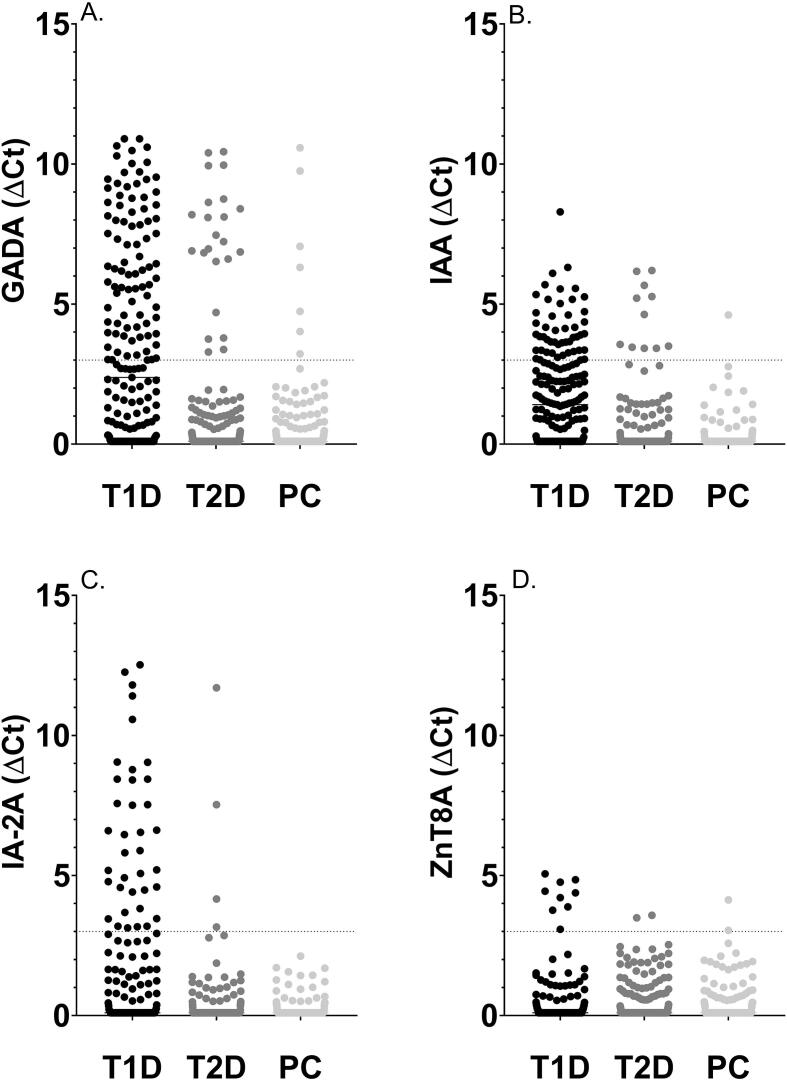
Scatter plots illustrating the prevalence of islet autoantibodies, GADA, IAA, IA-2A and ZnT8A in groups of T1D and T2D individuals, and population controls; A) GADA were detected in 46.1 % (88/191) of T1D individuals, 6.2 % (23/372) of T2D individuals and 2.6 % (7/259) of population controls (p^T1D_T2D^ < 0.0001, p^T1D_CTRLS^<0.0001, p^T2D_CTRLS^=0.0367), B) Prevalence of IAA were 47.1 % (90/191) in T1D individuals, 4.84 % (18/372) in T2D individuals and 2.32 % (6/259) in population controls (p^T1D_T2D^<0.0001, p^T1D_CTRLS^<0.0001, p^T2D_CTRLS^=0.10), C) IA-2A were reported in 22.5 % (43/191) of T1D individuals, 1.61 % (6/372) of T2D individuals and 0 % (0/259) of population controls (p^T1D_T2D^<0.0001, p^T1D_CTRLS^<0.0001, p^T2D_CTRLS^=0.09, D) Prevalence of ZnT8A was reported to 4.7 % (9/191) among T1D individuals, 0.54 % (2/272) among T2D individuals and 0.77 % (2/259) among population controls (p^T1D_T2D^=0.0014, p^T1D_CTRLS^=0.0108, p^T2D_CTRLS^>0.99. Autoantibodies were analyzed using ADAP assays. Autoantibody levels were presented as ΔCt, defined by the Cycle termination (Ct) value in the qPCR, of the average blank (phosphate-buffered saline and Triton-X) in the run subtracted by the Ct value of the sample. T1D; type 1 diabetes; T2D; type 2 diabetes; PC; population controls; GADA; glutamic acid decarboxylase autoantibodies; IAA; insulin autoantibodies; IA-2A; insulinoma antigen-2 autoantibodies; ZnT8A; zinc transporter 8 autoantibodies.

**Supplementary Fig. 1:**
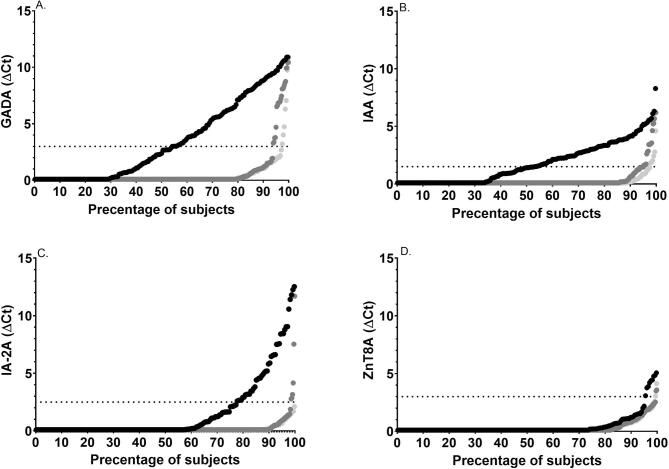


GADA, IAA, IA-2A and ZnT8A were detected in 46.1 %, 47.1 %, 22.5 % and 4.7 %, respectively, of individuals with T1D, Fig. 1 **and** Supplementary Fig. 1.

**Supplementary Fig. 2:**
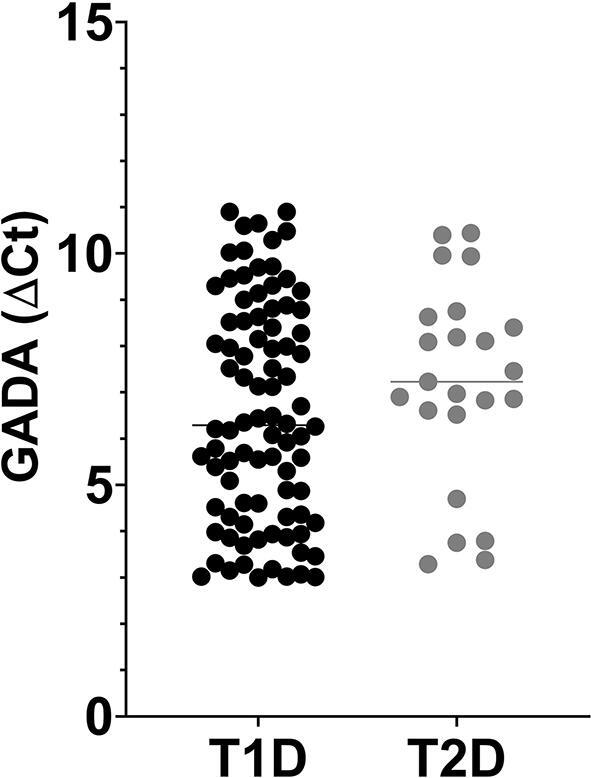


Prevalence of IAA (4.84 %, 2.32 %), IA-2A (1.61 %, 0 %) and ZnT8A (0.54 %, 0.77 %) was comparable between individuals with T2D and population controls (p = 0.10, p = 0.09, p > 0.99). GADA positivity, 6.2 %, was increased among individuals with T2D compared with population controls, 2.6 % (p = 0.0367), Fig. 1 **and** Supplementary Fig. 1. Islet autoantibody positivity was increased among individuals with T2D, 10.2 %, compared to population controls, 5.0 %, (p = 0.02185). The median body mass index was not different in GADA positive (27.2) and GADA negative individuals (28.4, p = 0.16). Likewise, median age was not different in GADA positive (67 years) and GADA negative individuals (71 years, p = 0.80). The GADA levels were not different among positive T1D and T2D individuals (p = 0.25), Supplementary Fig. 2.

**Fig. 2:**
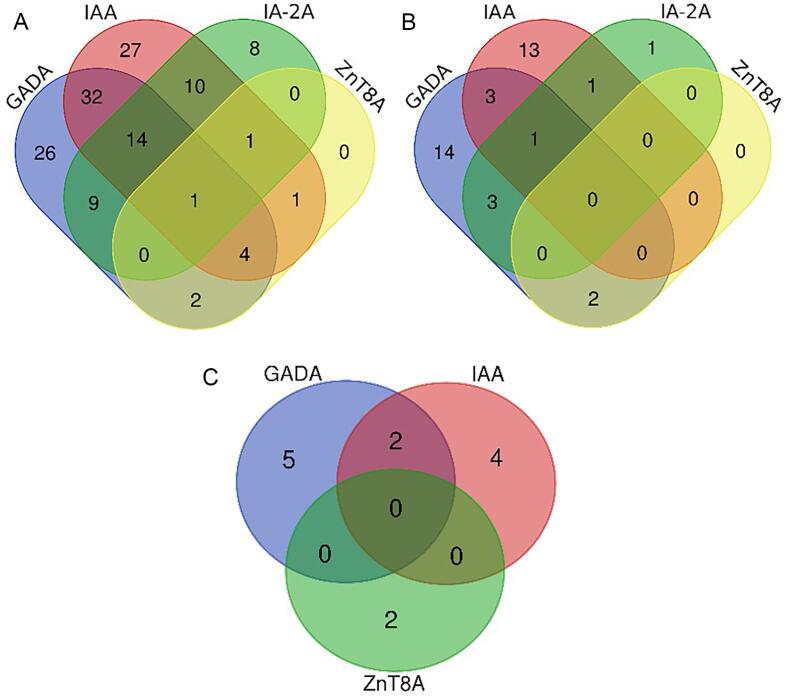
Venn diagrams illustrating the islet autoantibody profiles in A) T1D individuals, B) T2D individuals, C) Population controls T1D; type 1 diabetes; T2D; type 2 diabetes; PC; population controls; GADA; glutamic acid decarboxylase autoantibodies; IAA; insulin autoantibodies; IA-2A; insulinoma antigen-2 autoantibodies; ZnT8A; zinc transporter 8 autoantibodies.

The pattern of multiple islet autoantibody positivity in individuals with T1D, individuals with T2D and population controls is illustrated in Fig. 2A-C. We report 70.7 % of all individuals with T1D to be islet autoantibody positive, and of these individuals 31.9 % to be single positive and 38.7 % to be multiple positive.

### CD related autoantibodies

**Fig. 3:**
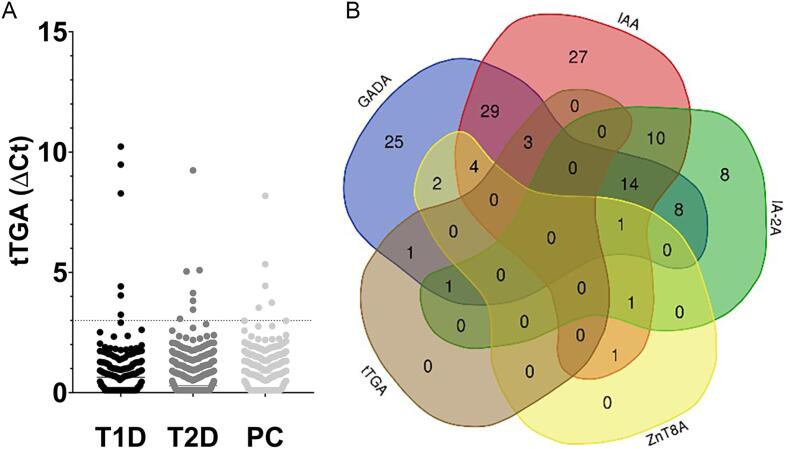
Tissue transglutaminase autoantibodies (tTGA) were analyzed in groups of T1D and T2D individuals, and population controls. Illustrated in: A) Scatter plot demonstrating detection of tTGA in 2.6 % (5/191) of T1D individuals, 1.2 % (4/372) of T2D individuals and 1.1 % (3/259) of population controls (p^T1D_T2D^=0.18, p^T1D_CTRLS^=0.29, p^T2D_CTRLS^>0.99), and B) Venn diagram demonstrating tTGA in association with islet autoantibodies in T1D individuals. Autoantibody levels in ADAP assays were presented as ΔCt, defined by the Cycle termination (Ct) value, of the average blank in the run subtracted by the Ct value of the sample. T1D; type 1 diabetes; T2D; type 2 diabetes; PC; population controls; tTGA; tissue transglutaminase autoantibodies; GADA; glutamic acid decarboxylase autoantibodies; IAA; insulin autoantibodies; IA-2A; insulinoma antigen-2 autoantibodies; ZnT8A; zinc transporter 8 autoantibodies.

**Supplementary Fig. 3:**
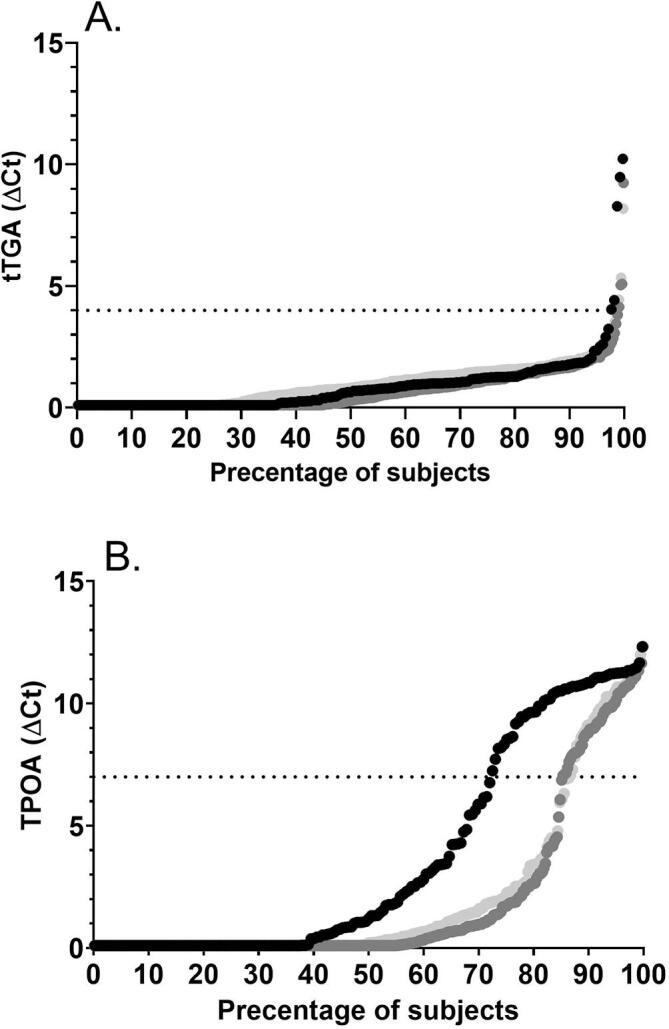


tTGA positivity was not different in individuals with T1D, 2.6 %, individuals with T2D, 1.2 %, and population controls, 1.1 % (p^T1D_T2D^=0.18, p^T1D_CTRLS^=0.29, p^T2D_CTRLS^ p > 0.99). In total 5 individuals with T1D were tTGA positive, of whom all were GADA positive. tTGA positivity in individuals with T2D and population controls was demonstrated in islet autoantibody negative individuals see Fig. 3 **and** Supplementary Fig. 3.

### AITD related autoantibodies

**Fig. 4:**
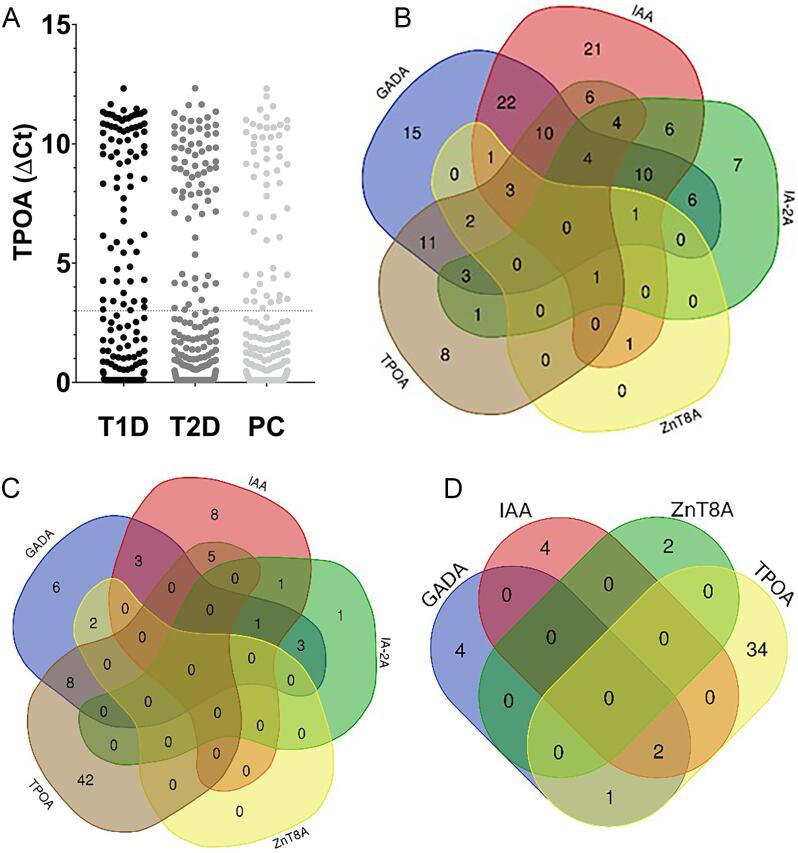
Thyroid peroxidase autoantibodies were analyzed in groups of T1D and T2D individuals, and population controls. Illustrated in: A) Scatter plot demonstrating detection of TPOA in 27.8 % (53/191) of T1D individuals, 14.8 % (55/372) of T2D individuals and 14.3 % (37/259) of population controls (p^T1D_T2D^=0.0002, p^T1D_CTRLS^=0.0004, p^T2D_CTRLS^=0.86), and venn diagrams demonstrating TPOA in association with islet autoantibodies in: B) T1D individuals, C) T2D individuals, and D) Population controls. Autoantibody levels were presented as ΔCt, defined by the Cycle termination (Ct) value, of the average blank in the run subtracted by the Ct value of the sample. T1D; type 1 diabetes; T2D; type 2 diabetes; PC; population controls; TPOA; thyroid peroxidase autoantibodies; GADA; glutamic acid decarboxylase autoantibodies; IAA; insulin autoantibodies; IA-2A; insulinoma antigen-2 autoantibodies; ZnT8A; zinc transporter 8 autoantibodies.

An increased prevalence of TPOA was demonstrated among individuals with T1D, 27.8 %, compared to individuals with T2D, 14.8 %, and population controls, 14.3 %, p^T1D_T2D^=0.0002, p^T1D_CTRLS^=0.0004, p^T2D_CTRLS^=0.86). TPOA was predominantly related to islet autoantibody positivity in individuals with T1D, 84.9 % (45/53), compared to individuals with T2D, 23.6 % (13/55), and population controls, 8.1 % (3/37), (p^T1D_T2D^<0.0001, p^T1D_CTRLS^<0.0001, p^T2D_CTRLS^ p = 0.09), Fig. 4**, and** Supplementary Fig. 3. TPOA was more frequent in GADA positive, 34.8 % (8/23), compared to GADA negative, 13.5 % (47/349) in individuals with T2D (p = 0.0053). Among GADA positive individuals, the levels were increased in TPOA positive compared to negative group for T2D (p = 0.0167) but not for T1D (p = 0.67), Supplementary Fig. 2.

## Discussion

We report an increased prevalence of GADA in individuals living with T2D compared to matched population controls. Among these GADA positive T2D individuals, TPOA was also increased, supporting the view that GADA is a biomarker of autoimmune type 2 diabetes. Our data and conclusion are consistent with the report that LADA was associated with TPOA, but not tTGA [18]. In addition, the prevalence of islet autoantibodies in our study was comparable to previous reports in T2D individuals classified or not with LADA [19], [20], [21].

Increased prevalence of GADA in T2D has been reported previously [22], however, if single islet autoantibody positivity is evidence of ongoing autoimmunity has been debated;

1) Arguments against T2D autoimmunity include the notion that islet autoantibodies may fluctuate, and may be transient in a preceding stage prior to T2D especially in younger individuals [23]. The presence of GADA is acknowledged in approximately 2 % of the general population without association to progressive autoimmunity, these natural autoantibodies are found in low quantities and may be of importance for immune responses and regulation [24]. Geographical variations in the presence of islet autoantibodies should be acknowledged [25] with lower autoantibody rates reported from African countries [26], [27], [28], [29], [30], [31]. It is therefore possible that autoantibodies are not a global marker for Beta cell autoimmunity, this may be increasingly reflected in the heterogenous appearance of T2D.

2) Reasonings supporting T2D autoimmunity include the notion that autoimmune processing could be masked by heterogeneity in T2D, and while there have been efforts to cluster adult-onset diabetes into subgroups [32], it is not clear where autoimmune T2D individuals belong. If islet autoimmunity precedes the onset of T2D, as indicated by the presence of autoreactive T cells in both islet autoantibody positive and negative T2D individuals [33], [34], [35], this may indicate an autoimmune triggering mechanism. In this case, GADA may remain a reliable biomarker for classification and risk stratification of T2D. The predictive value of GADA in T2D was emphasized in a study including non-diabetes individuals over the age of 40, which revealed that GADA positivity at baseline associated with diabetes diagnosis during 10-year follow-up [36]. Weaker association to T1D related HLA-risk genotypes in T2D individuals [20], [37] may be due to the fact that GADA appear following the onset of T2D.

Thyroid dysfunction has been increasingly associated with T2D [38], however, not previously in conjunction with GADA. Higher levels of GADA have been associated with thyroid autoimmunity in T1D. An equivalent relationship in GADA positive T2D individuals would imply benefits of autoantibody analyses to increase the diagnostic precision of individuals who have been classified and treated as T2D [39]. The contribution of GADA and TPOA to the pathogenesis of T2D needs to be investigated more thoroughly and may have clinical implications, for example longitudinally greater weight loss in obese individuals [19].

Beta cell function may differ in GADA positive compared to negative T2D individuals as both fasting C-peptide and insulin response to oral glucose is lower [37]. GADA positivity has been demonstrated to be associated with a faster progression to insulin deficiency [40]. The “accelerator hypothesis” suggests that there is an overlay rather than an overlap between the etiologies of T1D and T2D, with the rate of beta cell loss and the responsible accelerators for the diminishing beta cell mass as the distinguishing factors between the disease subtypes [41].

A similar theory with an overlay rather than overlap could be attributed to an increased prevalence of TPOA in both islet autoantibody positive T1D and GADA positive T2D individuals. Our findings may therefore suggest a shared mechanism of autoimmune nature between subtypes of diabetes. The smaller group of GADA and TPOA positive T2D individuals may be the focus for increased understanding of autoantibody positive non-progression in T1D diabetes in case these individuals should not progress to LADA or adult T1D.

The overlap between T1D and T2D was previously described to include a reverse correlation between systemic immune mediators and presence of autoantibodies. The demonstration of a graded change in immunological markers was interpretated as a reflection of shared pathological mechanisms between T1D and T2D [42]. The presence of TPOA in both T1D and T2D was reported in our study and previously [42], and TPOA may, therefore, be utilized as an analyte which can add mechanistical understanding of the overlap between the disease subtypes.

Our study did not relate a higher BMI to presence of GADA in T2D. Insulin resistance is a known complication of obesity, and there are reports of autoimmune response against the beta cells among obese children and adults, in the latter group also an association to TPOA has been observed [43], [44].

Higher GADA levels among TPOA positive compared to TPOA negative T2D individuals were in line with the literature [45], and could further support autoimmunity in a subgroup of T2D individuals. The relevance of GADA in T2D clinical phenotypes was previously investigated to suggest an association between higher levels and extended autoimmunity [45]. It would be of interest to expand these investigations further in T2D since GADA levels among T1D individuals has been acknowledged to improve stratification of autoimmune progression [46] and clinical presentation at time of diagnosis [47].

Our study did not relate advanced age to presence of GADA in T2D. Advancing age (above 65 years) could associate with increased GADA prevalence in T2D as a result of prolonged disease time [48], [49], or a weakened immunological response with increasing age could instead associate GADA with younger age (below 65 years) [50].

Strengths of the study included the approach to reach T2D and T1D as well as control individuals through registries and not by the individuals being approached at a hospital or clinic. In Sweden, most T2D individuals are cared for at primary health care centers. GADA analyses are not routine in primary health care which may explain the high prevalence of GADA among T2D individuals.

Our study included several limitations. The sample size was small given the heterogeneity in T1D and T2D populations. Data was missing on diabetes disease duration and clinical characteristics for individuals with both T1D and T2D. Our study included home-capillary sampling on a single occasion, and we could therefore not confirm persistent autoantibody positivity. The recruitment of study participants took place outside the healthcare system, a lower number of the total invite participated, a selection bias is not expected but cannot be fully excluded.

T2D is a heterogeneous disease, we add to this diversity by reporting of a GADA positive subgroup of T2D individuals with increased prevalence of TPOA. Combined GADA and TPOA analyses could refine the autoimmune landscape in individuals clinically classified with T2D.

## Funding

The studies were funded by NIH SBIR 2R44DK110005-02, The Strategic Research Area Exodiab (Dnr 2009-1039), and The Swedish Foundation for Strategic Research (Dnr IRC15-0067). The funding source(s) was not involved in study design, the collection, analysis and interpretation of data or the writing of the manuscript.

## CRediT authorship contribution statement

**Lind Alexander:** Conceptualization, Formal analysis, Investigation, Writing – original draft, Writing – review & editing, Visualization. **Tsai Cheng-ting:** Methodology, Software, Resources, Data curation, Writing – review & editing, Funding acquisition. **Lernmark Åke:** Conceptualization, Formal analysis, Writing – review & editing, Supervision. **Jendle Johan:** Conceptualization, Formal analysis, Resources, Writing – review & editing, Supervision, Project administration.

## Declaration of competing interest

The authors declare the following financial interests/personal relationships which may be considered as potential competing interests: JJ has received consultant- or lecture fees from Abbot, Astra Zeneca, Boehringer Ingelheim, Eli Lilly, Medtronic, Nordic Infucare, Novo Nordisk, and Sanofi. ÅL is a member of the Scientific Advisory Board of Diamyd Medical.

## Figures and Tables

**Supplementary Fig. 4:**